# Was ist gesichert in der Vorsorge des kolorektalen Karzinoms?

**DOI:** 10.1007/s00108-025-01983-5

**Published:** 2025-09-11

**Authors:** Michael Bretthauer

**Affiliations:** 1 Clinical Effectiveness Research Group, Institute of Clinical Medicine, University of Oslo, Gaustad Hospital, Building 20, Sognsvannsveien 21, 0372 Oslo, Norwegen; 2 Department of Transplantation Medicine, Oslo University Hospital, Oslo, Norwegen

**Keywords:** Koloskopie, Stuhltest auf okkultes Blut, Darmkrebs/Screening, Vorsorgeuntersuchungen/Komplikationen, Darmperforation, Colonoscopy, Fecal occult blood test, Colorectal cancer/Screening, Cancer prevention/complications, Intestinal perforation

## Abstract

**Hintergrund:**

Trotz fallender Inzidenz und Mortalität ist Darmkrebs (kolorektales Karzinom) in Deutschland immer noch die zweithäufigste Krebserkrankung bei Frauen (nach Brustkrebs) und die dritthäufigste bei Männern (nach Prostata- und Lungenkrebs). Darmkrebs eignet sich gut für Vorsorge oder Früherkennung mittels Screening. Die zwei vornehmlich empfohlenen Tests sind die Koloskopie und der fäkale immunchemische Test (FIT; Stuhltest auf okkultes Blut).

**Ziel der Arbeit (Fragestellung):**

Der vorliegende Beitrag erklärt wichtige konzeptuelle und praktische Unterschiede der angebotenen Screeningtests in Deutschland und beschreibt die neueste Evidenzlage für den Nutzen der am häufigsten angewendeten Screeningmethoden. Ziel des Beitrags ist es, Ärzten Entscheidungshilfen zu geben für Patienten, die sich für das Darmkrebsscreening interessieren.

**Material und Methoden:**

Gesicherte qualitativ hochwertige Evidenz aus randomisierten Studien zum Nutzen des Darmkrebsscreenings in Bezug auf Inzidenz und Mortalität des kolorektalen Karzinoms.

**Ergebnisse:**

Das Lebenszeitrisiko in Deutschland, an Darmkrebs zu erkranken, beträgt derzeit 5 % bei Frauen und 6,5 % bei Männern. Laut einer großen randomisierten Studie reduziert eine Vorsorgekoloskopie das Risiko von Darmkrebs über 10 Jahre von 1,2 % auf ungefähr 0,8–0,9 %. Neue Evidenz einer großen spanischen randomisierten Studie zeigt, dass der Nutzen eines FIT-Screenings jedes zweite Jahr als gleichwertig zur einer Koloskopie über 10 Jahre einzuordnen ist. Das Risiko einer Perforation während einer Koloskopie liegt bei ungefähr 0,01 %, und das Risiko einer Blutung bei 0,1 %.

**Schlussfolgerung:**

Bei der ärztlichen Patientenaufklärung zum Screening wird empfohlen, die oben genannten Zahlen für Nutzen und Risiko heranzuziehen und zu erklären.

## Abstract (en)

**Background:**

The incidence and mortality of colorectal cancer in Germany has declined in recent years. Nevertheless, colorectal cancer is still the second most common cancer in women (after breast cancer) and the third most common in men in Germany (after prostate cancer and lung cancer). Screening for colorectal cancer is well-established in many countries. The two most recommended screening strategies are colonoscopy and fecal immunochemical testing (FIT, stool test for occult blood).

**Objective:**

This article explains important conceptual and practical differences between the two strategies offered in Germany and summarizes the latest high-quality evidence for the benefits of the most frequently used screening tests. The aim is to provide physicians with decision aids for patients who show interest in colorectal cancer screening.

**Material and methods:**

Confirmed high-quality evidence from randomized trials on the benefits of screening for colorectal cancer with respect to incidence and mortality.

**Results:**

The lifetime risk to develop colorectal cancer in Germany is 5% for women and 6.5% for men. According to a large randomized trial, a screening colonoscopy reduces the risk of colorectal cancer from 1.2% to 0.8–0.9% after 10 years. New high-quality evidence from a recent Spanish randomized trial also showed that the benefits of FIT screening every other year are comparable to those of 1 colonoscopy over 10 years. The risks of perforation and bleeding during colonoscopy are 0.01% and 0.1%, respectively.

**Conclusion:**

During the medical patient clarification on screening, it is recommended that the abovenamed facts on benefits and risks should be included and explained.

In den letzten Jahren ist die Inzidenz von Darmkrebs (kolorektales Karzinom) in Deutschland um fast ein Viertel gefallen, auf knapp unter 60.000 Neuerkrankungen pro Jahr [1]. Die Mortalität des kolorektalen Karzinoms ist gleichfalls deutlich gesunken. Darmkrebs ist allerdings immer noch die zweithäufigste Krebserkrankung bei Frauen (nach Brustkrebs) und die dritthäufigste bei Männern (nach Prostata- und Lungenkrebs; [2]). Das Risiko, im Laufe des Lebens an Darmkrebs zu erkranken, beträgt in Deutschland etwa 5 % für Frauen und 6,5 % für Männer. Etwa zwei Drittel aller kolorektalen Karzinome treten im Kolon auf, ein Drittel im Rektum [2].

Das kolorektale Karzinom eignet sich im Vergleich zu vielen anderen Krebserkrankungen sehr gut für Screeningmaßnahmen. Im Gegensatz zu Brustkrebs oder Prostatakrebs, bei denen es keine eindeutigen benignen Vorstadien der Krebserkrankung gibt, ist die Bedeutung kolorektaler Polypen als Vorstadium von Darmkrebs hinreichend bekannt – vornehmlich betrifft dies Adenome, aber auch die weniger häufigen sogenannten serratierten Polypen. In geübter Hand ist die Koloskopie eine außerordentlich gute Methode zur Diagnose und Therapie kolorektaler Polypen. Deutschland hat schon seit einigen Jahrzehnten ein opportunistisches Screeningangebot mit Stuhltests. Ab 2002 wurde auch die Koloskopie als primäre Screeninguntersuchung in Deutschland opportunistisch angeboten. Seit 2019 gibt es nun in Deutschland ein organisiertes Krebsfrüherkennungsprogramm für Darmkrebs. Anspruchsberechtigte Versicherte ab einem Alter von 50 Jahren erhalten von ihrer Krankenkasse eine Einladung zum Darmkrebsscreening.

Seit 2019 gibt es in Deutschland ein organisiertes Screeningsprogramm für Darmkrebs

Inwieweit das Darmkrebsscreening für die fallende Inzidenz und Mortalität der Erkrankung verantwortlich ist, ist schwer zu beurteilen. Es ist naheliegend, die positive epidemiologische Entwicklung der Erkrankung zumindest teilweise den Screeningmaßnahmen der letzten Jahre in Deutschland zuzuordnen [1]. Allerdings werden auch in Ländern, in denen bis vor Kurzem kein Screening angeboten wurde, fallende Risikoraten beobachtet, sodass die exakte Quantifizierung des Beitrags des Screenings zur erfreulichen Entwicklung herausfordernd ist [3].

Die allgemein anerkannte Evidenzpyramide in der klinische Medizin beschreibt den Stellenwert der vorliegenden Daten zu Nutzen und Risiko von Medikamenten und anderen medizinischen Tests und Interventionen [4]. Als die am besten gesicherte Evidenz werden randomisierte Studien und Metaanalysen dieser Studien angesehen. Die Evidenzlage zum Nutzen der zwei am häufigsten angebotenen Screeningtests in Deutschland – der Koloskopie und des Screening mit Stuhltests (fäkale immunchemische Tests [FIT]) – war bis vor Kurzem nicht gesichert, weil keine randomisierten Studien vorlagen, die den Nutzen dieser Screeningmethoden hinsichtlich Inzidenz und Mortalität des kolorektalen Karzinoms untersuchten. Das hat sich mittlerweile geändert, da 2022 und 2025 zwei große randomisierte Studien veröffentlicht wurden, die die Evidenzlage deutlich verbessert haben.

Der vorliegende Beitrag erklärt wichtige konzeptuelle und praktische Unterschiede der angebotenen Screeningtests in Deutschland und beschreibt die neue Evidenzlage für den Nutzen der zwei am meisten angewendeten Screeningmethoden. Ziel des Beitrags ist es, Ärzten Entscheidungshilfen zu geben für Patienten, die sich für Darmkrebsvorsorge interessieren und eine Teilnahme erwägen.

## Vorsorge und Früherkennung

Krebsscreening umfasst zwei sehr unterschiedliche Ansätze, die es gilt, deutlich zu unterscheiden, um falsche Erwartungen bei Entscheidungsträgern, Patienten und Öffentlichkeit zu verhindern. Der erste und ursprüngliche Ansatz ist die sogenannte Krebsfrüherkennung. Beispiele sind die Mammographie zur Detektion von Brustkrebs, das computertomographische Lungenkrebsscreening bei Rauchern und das Hautkrebsscreening. Die meisten heute angebotenen Screeningtests fallen in diese Kategorie. Der andere Ansatz ist die sogenannte Krebsvorsorge. Die zwei Vorsorgetests, die heute angeboten werden, sind das endoskopische Screening für das kolorektale Karzinom (Koloskopie, früher auch Sigmoidoskopie) und das Gebärmutterhalsscreening mit Papanicolaou(Pap)-Abstrich oder Test auf humane Papillomaviren (HPV).

In der Krebsfrüherkennung können nur Tumoren entdeckt werden, die schon invasiv wachsen

In der Krebsfrüherkennung können nur Tumoren entdeckt werden, die schon invasiv wachsen. Krebsvorsorgetests zielen dagegen vornehmlich darauf ab, noch benigne Vorstadien zu entdecken, die dann entfernt werden können. Dies bedeutet, dass Krebsfrüherkennung das Risiko, an Krebs zu erkranken, nicht reduzieren kann. Oft führt die Krebsfrüherkennung sogar zur Zunahme von Krebsdiagnosen (sogenannte Überdiagnostik). Das Ziel der Krebsfrüherkennung ist, die Prognose der diagnostizierten Patienten zu verbessern, um dadurch die Mortalität der Erkrankung zu senken. Krebsvorsorge dagegen, wie etwa bei der Koloskopie, zielt darauf ab, das Risiko zu senken, an Krebs an sich zu erkranken. Eine Konsequenz ist dann die Senkung der Mortalität aufgrund des logischen Sachverhalts, dass nur ein an Krebs erkrankter Patient auch an der Krebserkrankung sterben kann. Ein Patient, der keinen Krebs entwickelt, stirbt auch nicht daran.

## Screeningtests

Die Screeningtests für das kolorektale Karzinom können in zwei Gruppen eingeteilt werden: Stuhltests und endoskopische Verfahren.

### Stuhltests

Der vornehmlich angewendete Stuhltest ist der FIT, der Blut im Stuhl detektiert. Der FIT ist der am meisten eingesetzte Test in europäischen Ländern, wo er entweder in einem opportunistischen Screening über den Hausarzt oder in einem organisierten Screeningprogramm des Gesundheitsträgers angeboten wird. Da Stuhltests eine relativ geringe Sensitivität für Frühstadien von Darmkrebs haben, wird empfohlen, den Stuhltest jedes zweite Jahr zu wiederholen. Der Gedanke ist, dass die relativ geringe Sensitivität über das Wiederholen des Tests erhöht wird und somit ein Tumor, der bei einem Test nicht detektiert wurde, in zwei Jahren erkannt werden kann, wenn der Test wiederholt wird.

Das FIT-Screening ist vornehmlich in die Kategorie der Krebsfrüherkennung einzuordnen

Das FIT-Screening ist vornehmlich in die Kategorie der Krebsfrüherkennung einzuordnen, da die benignen Vorstadien des kolorektalen Karzinoms, also Polypen, oft nicht bluten und deswegen nicht gut mithilfe eines FIT entdeckt werden können. Deswegen hat das Stuhlscreening auch nur eine geringe Wirkung auf die Inzidenz des kolorektalen Karzinoms [4]. Ein FIT-Screeningprogramm kann allerdings in Richtung Krebsvorsorge verschoben werden, wenn man den Grenzwert eines positiven Tests sehr gering hält, weil dann viele der Menschen zur Koloskopie überwiesen werden.

### Endoskopische Methoden

Seit 20 Jahren ist die Koloskopie die dominierende Methode für das Screening auf kolorektale Karzinome in Deutschland. Früher wurde auch die Sigmoidoskopie angeboten, die aber mit der Zeit an Bedeutung verloren hat. Die Koloskopie wird als primärer Screeningtest in nur wenigen Ländern angeboten (Deutschland, USA, Österreich). Polen hatte bis vor einigen Jahren ein Koloskopiescreeningprogramm, ist aber vor Kurzem zu FIT-Stuhltests übergegangen.

Ein Primärscreening mit Koloskopie zielt vornehmlich darauf, benigne Polypen zu entdecken, bevor sie sich zu Krebs entwickeln, und diese während der Koloskopie zu entfernen. Weil die Zeitspanne, in der sich ein Polyp möglicherweise zum invasiven Tumor entwickelt, mit 10–15 Jahren sehr lang ist, wird die Koloskopie mit sehr viel längeren Zeitintervallen angeboten als die FIT-Stuhltests. In Deutschland liegt das empfohlene Screeningintervall der Koloskopie bei 10 Jahren.

## Mortalität und Inzidenz – Koloskopie und fäkale immunchemische Tests

Bis vor Kurzem gab es nur nichtgesicherte, indirekte Evidenz für den Nutzen von Koloskopie- und FIT-Screenings in Bezug auf Inzidenz und Mortalität des kolorektalen Karzinoms. In den letzten drei Jahren sind allerdings zwei große randomisierte Studien veröffentlicht worden, die die Evidenzlage wesentlich verbessern [6, 7].

### NordICC-Studie

Die NordICC-Studie ist eine große Multicenterstudie, in der fast 100.000 Menschen im Alter von 55 bis 64 Jahren in vier europäischen Ländern (Polen, Norwegen, Niederlande und Schweden) zum Koloskopiescreening oder Nichtscreening randomisiert wurden. In den Studiengebieten gab es zur Zeit der Studie kein Screening, sodass eine Kontrollgruppe ohne Screening etabliert werden konnte, in der die Menschen keinen Zugang zum Screening hatten. Die Ergebnisse der Studie wurden im Oktober 2022 im *New England Journal of Medicine* publiziert. Die NordICC-Studiengruppe verglich hier Inzidenz und Mortalität des kolorektalen Karzinoms nach 10 Jahren in der Screeninggruppe und der Nichtscreeninggruppe [5].

Das Risiko, am kolorektalen Karzinom zu erkranken, betrug 0,98 % bei Menschen in der Koloskopiegruppe und 1,2 % in der Nichtscreeninggruppe. Dies bedeutet eine relative Risikoreduktion von 18 %. Bei der Mortalität des kolorektalen Karzinoms war kein signifikanter oder klinisch bedeutsamer Unterschied zu beobachten (0,28 % und 0,31 % in den beiden Gruppen; [6]).

In der NordICC-Studie wurden alle Menschen, die in den Studiengebieten wohnten, randomisiert (sogenannte Populationsstudie). Da natürlich nicht alle Personen, die zum Koloskopiescreening randomisiert wurden, die Einladung wahrgenommen haben (ungefähr 40 % haben das Angebot angenommen und eine Koloskopie durchführen lassen), analysierte das NordICC-Team auch den Koloskopieeffekt bei denen, die tatsächlich teilgenommen haben, im Vergleich zur Nichtscreeninggruppe. Diese sogenannte Per-Protokoll-Analyse liefert keine gesicherte Evidenz, weil es schwierig ist, statistische Unterschiede zwischen den Gruppen analytisch auszugleichen. Mit diesen Vorbehalten waren die Ergebnisse wie folgt: Die Inzidenz des kolorektalen Karzinoms betrug 1,22 % in der Nichtscreeninggruppe und 0,84 % bei denen, die eine Koloskopie erhielten. Das entspricht einer relativen Reduktion der Inzidenz von 31 %. Die Mortalität war bei den Screeningteilnehmern 50 % niedriger als in der Nichtscreeninggruppe: 0,15 % und 0,3 % starben in den beiden Gruppen innerhalb von 10 Jahren an Darmkrebs.

### COLONPREV-Studie

Im April 2025 wurde die sogenannte COLONPREV-Studie in der Zeitschrift *The Lancet* publiziert [7]. Diese große randomisierte Studie verglich das Koloskopiescreening mit dem FIT-Screening in Spanien. Es ist die erste Studie dieser Art, die die zwei vornehmlich angebotenen Tests direkt vergleicht. Wie die NordICC-Studie war auch diese Studie populationsbasiert. In der Studie wurden 57.000 Menschen zwischen 50 und 69 Jahren in 13 spanischen Regionen randomisiert. Der einen Gruppe wurde eine Koloskopie angeboten und der anderen Gruppe ein FIT-Stuhltest, der jedes zweite Jahr wiederholt wurde.

Nach 10 Jahren Nachbeobachtung in beiden Gruppen betrug das Risiko, an einem kolorektalen Karzinom zu versterben, 0,22 % in der Koloskopiegruppe und 0,24 % in der FIT-Gruppe. Das heißt, dass kein Unterschied in der Mortalität zwischen Koloskopiescreening und FIT-Screening beobachtet wurde. Die Inzidenz von kolorektalen Karzinomen war in der Koloskopiegruppe (1,13 %) nur geringfügig kleiner als in der FIT-Gruppe (1,22 %). Die Teilnehmerrate in den beiden Gruppen war kleiner als in der NordICC-Studie, aber in den beiden Gruppen ungefähr gleich; 20 % in der Koloskopiegruppe und zwischen 18 und 30 % in den unterschiedlichen Screeningrunden der FIT-Gruppe [8]. Das heißt, die Ergebnisse sind in der Tat vergleichbar.

Auch im Rahmen der COLONPREV-Studie wurden Per-Protokoll-Analysen zum Vergleich der Koloskopie mit dem FIT-Screening publiziert (Anhang des *Lancet*-Artikels). Wie auch bei der NordICC-Studie sind diese Analysen wegen der statistischen Schwierigkeiten mit Vorsicht zu genießen und liefern keine gesicherte Evidenz. Das Risiko, an einem kolorektalen Karzinom zu erkranken, betrug 0,85 % in der Koloskopiegruppe und 1,28 % in der FIT-Screening-Gruppe, und die Risiken, an einem kolorektalen Karzinom zu versterben, lagen bei 0,02 % und 0,11 % in den beiden Gruppen. Diese Unterschiede waren nicht statistisch signifikant und dürften wohl auch für die meisten Menschen keinen klinisch relevanten Unterschied darstellen.

## Verlängert Screening das Leben?

„Screening rettet Leben“ ist ein bekannter und oft benutzter Slogan von Krebshilfen und Patientenorganisationen sowie von einigen Gesundheitsträgern und Ärzten [9]. Der Slogan ist gute Werbung und erscheint für viele logisch. Um herauszufinden, ob Screening tatsächlich Leben rettet, muss man allerdings fragen, ob Menschen, die am Screening teilnehmen, länger leben als Menschen, die nicht am Screening teilnehmen.

Die beste Evidenzlage ergibt sich wie immer in der Medizin aus randomisierten Studien. Menschen, die am Screening teilnehmen, sind oft gesünder und haben ohnehin eine längere Lebenserwartung als Menschen, die nicht am Screening teilnehmen. Daher muss man, um gesicherte Daten zu erhalten, die Lebenslänge von Menschen vergleichen, die zu Screening oder Nichtscreening bzw. zu verschiedenen Screeningtests randomisiert wurden. Es bietet sich also an, die Ergebnisse der zwei oben erwähnten großen randomisierten Studien zur Gesamtsterblichkeit heranzuziehen.

- In der NordICC-Studie verstarben innerhalb der 10 Jahre insgesamt 11,03 % der Menschen in der Screeninggruppe und 11,04 % der Menschen in der Nichtscreeninggruppe, ein Unterschied von 0,1 %, der nicht statistisch signifikant ist.
- In der spanischen COLONPREV-Studie verstarben innerhalb von 10 Jahren 7,64 % der Menschen in der Koloskopiegruppe und 7,68 % der Menschen in der FIT-Gruppe, ein Unterschied von 0,4 %. Auch dieser Unterschied ist nicht statistisch signifikant.

Es ist also zurzeit nicht gesichert, dass das kolorektale Screening zu einer Steigerung der Lebenslänge in der Bevölkerung führt. Angesichts des relativ geringen Anteils der Sterblichkeit des kolorektalen Karzinoms an der Gesamtsterblichkeit ist dies nicht sehr überraschend. Die Ergebnisse sollten dennoch nicht als disqualifizierend für das kolorektale Screening eingeordnet werden. Aber vielleicht sollte auf Slogans mit Bezug zur Lebensrettung nach Möglichkeit verzichtet werden. Das Koloskopiescreening senkt die Inzidenz um ungefähr 30 %, und das kolorektale Screening an sich senkt wahrscheinlich die Sterblichkeit des kolorektalen Karzinoms. Das sollte zu Werbezwecken und zur Motivation genügen.

## Nachteile und Komplikationen

Alle medizinischen Behandlungen und Interventionen haben Vor- und Nachteile. Es gibt in der Medizin leider oft keinen Nutzen ohne Nachteil. Jede Koloskopie ist eine Belastung für den Patienten. Die Vorbereitung zur Koloskopie wird von vielen als unangenehm empfunden. Die Untersuchung an sich erfordert oft eine Sedierung, und Koloskopien bei nichtsedierten Patienten (in Deutschland die Ausnahme, in manchen anderen Ländern die Regel) sind oft mit Unbehagen oder Schmerzen verbunden. Die Kosten der Koloskopie für das Gesundheitswesen und für selbst zahlende Patienten sind nicht unerheblich.

Jede Koloskopie ist eine Belastung für den Patienten

Stuhltests sind an sich mit weniger Nachteilen und Kosten verbunden als die Koloskopie, sowohl für den Patienten als auch für den Kostenträger. Aber bei allen Menschen mit positivem Test muss natürlich eine Koloskopie durchgeführt werden, um am Nutzen der Vorsorge teilhaben zu können – und mit einem FIT jedes zweite Jahr, wie zurzeit empfohlen, ist die kumulative Wahrscheinlichkeit sehr groß, zumindest einmal positiv getestet zu werden; in der spanischen Studie beispielsweise fast 30 % [8]. Für einige Menschen ist es auch eine psychologische Belastung, jedes zweite Jahr einen Stuhltest durchzuführen und damit an die Erkrankung erinnert zu werden. Eine gewisse Müdigkeit, an repetitiven Stuhltests teilzunehmen, ist in der spanischen COLONPREV-Studie nachzuweisen. Die Teilnahme verringerte sich von über 30 % in der ersten FIT-Runde auf unter 20 % im Laufe der weiteren vier Runden [7].

Das Risiko von Komplikationen bei der Koloskopie, entweder als primäre Vorsorgeuntersuchung oder nach einem positiven FIT, ist klein. Komplikationen treten aber durchaus auf und können schwerwiegend sein. Das Risiko einer Perforation während einer Koloskopie liegt bei ungefähr 0,01 %, und das Risiko einer Blutung bei 0,1 % [10, 11].

## Zusammenfassung der Vor- und Nachteile

Wenn man das Risiko von Komplikationen (Perforation und Blutung) mit den oben genannten Zahlen zur Inzidenzreduktion vergleicht, ergibt sich ein zu erwartender Nutzen über 10 Jahre von etwa 0,3 % hinsichtlich des Risikos, an Darmkrebs zu erkranken, bei einem Risiko von Blutung oder Perforation von ungefähr 0,11 %. Die Zahlen sind hilfreich in einem Aufklärungsgespräch, müssen vom Arzt aber kontextuell aufgearbeitet werden, da die Verhinderung eines Karzinoms wahrscheinlich für die meisten Menschen einen höheren Wert beinhaltet als die Verhinderung einer Blutung während einer Koloskopie. Zusammen mit einer guten ärztlichen Erklärung und Einordnung können diese Zahlen bei einer Patientenaufklärung zum kolorektalen Screening sehr hilfreich sein.

Wie der Nutzen der Screeningtests sich nach mehr als 10-jähriger Beobachtungszeit entwickelt, wird die Zukunft zeigen. NordICC und COLONPREV werden die Teilnehmer weiter nachverfolgen und in einigen Jahren neue Daten veröffentlichen. Ähnliche Studien werden zurzeit auch in Schweden und den USA durchgeführt [12, 13]. Deren Ergebnisse werden für das Ende dieses Jahrzehnts erwartet.

## Screeningqualität

Die Koloskopie ist eine technisch schwierige Untersuchung, die viel Training und Übung erfordert. Es ist hinreichend bekannt, dass es große Unterschiede in der Qualität der Koloskopie zwischen ausführenden Ärzten gibt, auch in Deutschland [14]. Die Europäische Gesellschaft für gastrointestinale Endoskopie (European Society of Gastrointestinal Endoscopy [ESGE]) empfiehlt, eine Reihe sogenannter wichtiger Qualitätsmerkmale („key performance indicators“) bei jeder Koloskopie von jedem durchführenden Arzt zu erfassen [15].

Bei der Screeningkoloskopie ist die Adenomdetektionsrate das wichtigste Qualitätsmerkmal

Bei der Screeningkoloskopie ist die Adenomdetektionsrate (ADR) das wichtigste Qualitätsmerkmal. Bis vor Kurzem wurde eine möglichst hohe ADR angestrebt, ohne obere Grenze [15]. Neue qualitativ hochwertige Daten aus Polen zeigen, dass es für die Vorsorgequalität am wichtigsten ist, alle Endoskopiker, die Screeningkoloskopien durchführen, auf ein Mindestmaß der Adenomdetektion hin zu überwachen [16]. Dieser Schwellenwert liegt in vielen Ländern bei 25 % [17].

Die kontinuierliche Erfassung und Registrierung der ADR sollte für alle am Screening teilnehmenden Endoskopiker selbstverständlich sein. Nur so kann der Erfolg des kolorektalen Screenings in der Zukunft gesichert werden. Die gleichen Prinzipien und Maßnahmen gelten auch für die Koloskopie nach positivem FIT, wobei die angestrebten ADR-Raten hier höher sind [18].

## Fazit für die Praxis

- Inzidenz und Mortalität des kolorektalen Karzinoms sind in den letzten Jahren in Deutschland deutlich gesunken.
- Die in Deutschland empfohlenen Strategien zum Darmkrebsscreening sind die Koloskopie jedes zehnte Jahr und der fäkale immunchemische Test (FIT) auf okkultes Blut jedes zweite Jahr ab dem 50. Lebensjahr.
- Das Lebensrisiko in Deutschland, an Darmkrebs zu erkranken, beträgt derzeit 5 % bei Frauen und 6,5 % bei Männern.
- Neue gesicherte Evidenz einer großen randomisierten Studie in vier europäischen Ländern belegt, dass ein Koloskopiescreening das Risiko von Darmkrebs über 10 Jahre um ungefähr 30–50 % senken kann.
- Neue gesicherte Evidenz einer großen randomisierten Studie zeigt, dass der 10-jährige Nutzen des FIT-Screenings als gleichwertig zur Koloskopie einzuordnen ist.
- Das Risiko einer Perforation während einer Koloskopie liegt bei ungefähr 0,01 %, und das Risiko einer Blutung bei 0,1 %.
- Bei der ärztlichen Patientenaufklärung über das Screening wird empfohlen, die oben genannten Zahlen zu Nutzen und Risiko heranzuziehen und zu erklären.
